# Convergence of two serotypes within the epidemic ST11 KPC-producing *Klebsiella pneumoniae* creates the “Perfect Storm” in a teaching hospital

**DOI:** 10.1186/s12864-022-08924-8

**Published:** 2022-10-07

**Authors:** Chao Liu, Ping Yang, Jiajia Zheng, Juan Yi, Ming Lu, Ning Shen

**Affiliations:** 1 Department of Infectious Diseases, Peking University Third Hospital, Beijing, China; 2 Center of the Infectious Disease, Peking University Third Hospital, Beijing, China; 3 Institute of Medical Technology, Peking University Health Science Center, Beijing, China; 4 Department of Respiratory and Critical Care Medicine, Peking University Third Hospital, Beijing, China; 5 Department of Laboratory Medicine, Peking University Third Hospital, Beijing, China

**Keywords:** *Klebsiella pneumoniae*, ST11, Hypervirulence, Carbapenem resistance, Whole-genome sequencing

## Abstract

**Objectives:**

ST11 KPC-producing *Klebsiella pneumoniae* (Kp) is highly prevalent in China. We investigated the inter- and intra- host transmission and evolution characteristics of ST11 KPC-producing Kp.

**Methods:**

A retrospective study was conducted in a hospital. The clinical data and antimicrobial resistance (AMR) phenotypes were collected. Whole genome sequencing was performed. The transmission route was reconstructed by combining single nucleotide polymorphisms (SNPs) with the clinical information. Hypervirulent Kp (HvKp) was defined as the presence of some combination of *peg-344, iroB, iucA, rmpA*, or *rmpA2*.

**Results:**

Fifty-eight Kp strains isolated from thirty-five patients were enrolled. The information of one isolate was missing. The mean age of the patients was 74.3 ± 18.0 years, and 18 (50.0%) were female. Fifteen patients (41.7%, 15/36) presented with poor prognosis. All the strains were identified as ST11, and 57 strains harbored *bla*_KPC-2_. Two distinguished clades were identified based on the 1,325 high quality SNPs. In clade 1, carbapenem-resistant (CR)-hvKp accounted for 48.3% of the strains (28/58), which mostly presented as KL64 subclones, whereas CR-classical *Klebsiella pneumoniae* (cKp) commonly possessing KL47 were clustered in Clade 2. One CR-hvKp strain might have originated from the CR-cKp strain from within-host evolution. Even worse, a prolonged transmission of CR-hvKp has led to its spread into healthcare institutes.

**Conclusion:**

Two endemic subclones of ST11 KPC-producing Kp, KL64-CR-hvKp and KL47-CR-cKp, were transmitted in parallel within the hospital and/or the healthcare institute, suggesting that the ongoing genomic surveillance should be enhanced.

**Supplementary Information:**

The online version contains supplementary material available at 10.1186/s12864-022-08924-8.

## Introduction

*Klebsiella pneumoniae* (Kp) is increasingly emerging in China and has become the dominant pathogen in hospital-acquired infections (HAIs) [1]. Kp is a common cause of various fatal infections, posing a great threat to healthcare services [2]. Notably, Kp has evolved into two main pathotypes: hypervirulent *Klebsiella pneumoniae* (hvKp) and classical *Klebsiella pneumoniae* (cKp) [3, 4]. Previous studies demonstrated that hvKp is highly associated with community-acquired infection (CAI) and is sensitive to most antibiotics, whereas cKp is closely related to HAI and is resistant to most antimicrobial agents [3, 5].

Initially, the string test was used to distinguish hvKp and cKp [6]. However, some studies confirmed that the string test presented a lower prediction for identifying hvKp [7–9]. Recently, a combination of five virulence-associated genes, *peg-344, iroB, iucA, rmpA,* and *rmpA2*, showed higher diagnostic accuracy for hvKp than the string test or other virulence-associated genes [10, 11]. In our previous study, we found that hvKp replaced cKp as the dominant pathotype in the HAI, which primarily contributed to the acquisition of key virulence genes within the prevalent cKp (ST11) [11]. In China, the ST23 clone was common in the hvKp group, and ST11 was the prevalent cKp [1, 6, 12, 13]. ST11 cKp was highly associated with the Mulitdrug-resistance (MDR) or Carbapenem-resistance (CR) phenotype due to the acquisition of *bla*_KPC-2_ [1, 14]. The serotypes KL64 and KL47 were common within the ST11 Kp strain [11], but the epidemiological trends showed that KL64, not KL47, is prevalent among ST11 strains [15]. However, ST11 cKp is becoming hypervirulent by acquiring various virulence genes or a pLVKP-like plasmid [11, 16, 17]. These ST11 MDR hvKp strains have caused serious nosocomial infections and outbreaks worldwide [11, 17, 18]. Therefore, deeply understanding the genomic variation of ST11 during transmission might be a good choice to optimize surveillance.

Previous studies have mostly focused on the Kp strains isolated from different hosts. However, it is important to enroll the strains during in vivo evolution to construct the comprehensive transmission route by whole genome sequencing [19]. Therefore, our group conducted a retrospective study to clearly understand the genomic characteristics and evolution within the ST11 strains. Surprisingly, we found that ST11 has successfully evolved into two clades in which CR-hvKp that presented with KL64 and CR-cKp that was associated with KL47 were circulated in parallel within the hospital and the healthcare institute, suggesting that enhanced genomic surveillance is essential.

## Materials and methods

### Enrolled patients

A retrospective study was conducted from December 2017 to December 2020 at a hospital, and the isolates were selected. The distance between the branch of Peking University Third Hospital and headquarters is approximately 27 km. The clinical information of patients with ST11 infection was collected, including the basic demographic characteristics, admitted department, isolated time, specimen type, underlying diseases, antibiotic exposure within 90 days, Charlson comorbidity index (CCI), usage of invasive catheters and sequential organ failure assessment (SOFA). The presence of > 1 infection site in the same patient was defined as a metastatic infection. The infection types of the patients were divided into three groups: community-acquired infection, healthcare-associated infection (HCAI) and hospital-acquired infection (HAI). The definitions were as previously described [11]. The primary endpoint was poor prognosis within 30 days (death and withheld life-sustaining therapy).

The protocol for this study was approved by the Peking University Third Hospital Ethics Committee (M2021545). Due to the retrospective nature of the study, informed consent was not essential, and all the patient data in this study were anonymized.

### Clinical Klebsiella pneumoniae strains

All the Kp strains were stored at -80 °C. The strains were initially inoculated and cultured on Columbia blood agar. The monoclonal strain was selected for identification by MALDI-TOF mass spectrometry and then by the Vitek compact 2 system. The definition of hvKp was the presence of some combination of *peg-344, iroB, iucA, rmpA*, or *rmpA2* [10].

### Antimicrobial susceptibility testing (AST)

AST was performed by a Vitek 2 system (bioMérieux, Marcy-l’Étoile, France). If necessary, we also used the disk diffusion method. The results were interpreted according to the 2020 Clinical and Laboratory Standards Institute (CLSI) guidelines. The antibiotics used for AST included Imipenem (IPM), Meropenem (MEM), Ertapenem (ETP), Minocycline (MNO), Amikacin (AMK), Cefepime (FEP), Ceftazidime (CAZ), Levofloxacin (LVX), Piperacillin/tazobactam (TZP), Cefoperazone/sulbactam (CSL) and trimethoprim/sulfamethoxazole (SXT). Strains resistant to three or more different antimicrobial categories were defined as having a multidrug resistance (MDR) phenotype [12]. Additionally, the definition of carbapenem resistance (CR) was the presence of resistance to IPM, MEM, or ETP [20].

### DNA extraction and whole genome sequencing

Whole genomic DNA was extracted by the MagaBio Bacterium DNA Fast Purification Kit (BSC45S1E, Bioer Technology, Hangzhou, Zhejiang, China), and all strains were sequenced using the NovaSeq platform by constructing paired-end libraries to obtain 150 bp reads as previously described. We used fastQC software to obtain the clean data from the raw data and assembled the clean data by using SPAdes v3.15.2 [11]. Then, the draft genome sequences were annotated using Prokka [14].

To clearly determine the subtypes of these isolates, the sequencing type (ST) and capsular types were analyzed using Kleborate [21]. Antimicrobial resistance genes, virulence genes, and plasmid replicon types were annotated by comparison with relevant databases (ResFinder, Virulence Factor Database, plasmidFinder) using BLAST software. The thresholds of 90% identity and minimum length coverage of 80% for identifying antimicrobial resistance and virulence genes were determined.

To perform phylogenetic analysis, we applied the complete genome sequence of *K.pneumoniae* HS11286 (accession no.: NC_016845) as the reference. The sequencing reads were mapped to the reference using Bowtie 2 v2.2.8, and the single nucleotide polymorphisms (SNPs) were analyzed by using Samtools v1.9 and were combined together according to the reference using the iSNV-calling pipeline that we previously constructed [22, 23]. The high-quality SNPs supported by more than 5 reads of a mapping quality > 20 remained. Then, the recombination sites were detected by Gubbins [11, 24]. The concatenated sequences of the filtered polymorphic sites that were conserved in all the Kp strains (core genome SNPs, cgSNPs) were used to perform phylogenetic analysis using the maximum likelihood method by FastTree as previously described [11].

## Results

### Clinical characteristics of the ST11 Kp-infected patients

To perform a potential transmission route, Kp strains isolated from inter- and intrahosts were enrolled in the study. In total, fifty-eight Kp strains isolated from thirty-five patients were incorporated into this study. Four Kp strains were isolated from one of the 36 patients. Among seven patients, three isolates were detected from each patient, and two strains were obtained from each of the other six patients. Additionally, the information of one strain was missing.

One strain was isolated at December 2017, while three strains were isolated at September 2018, and the other strains were all from 2020 (Additional file 1: Figure S1). Most of the strains (56.9%, 33/58) were isolated from the respiratory tract. Fourteen strains (24.1%, 14/58) were from urine (Additional file 1: Figure S2).

The mean age of the enrolled patients was 74.3 ± 18.0 years, and 18 (50.0%) were female. Thirty-four patients were admitted to six departments of the headquarters, including the emergency department (17/36), ICU (9/36), geriatric ward (3/36), outpatient department (1/36), orthopedics department (1/36), cardiac surgery ward (1/36) and general surgery department (1/36). The other two patients were hospitalized in the branch of the hospital. Most of the patients presented with cardiovascular disease (32/36, 88.9%). More than half of the patients had cerebrovascular disease (20/36, 55.6%), and fourteen patients (38.9%, 14/36) had diabetes. Importantly, all patients had antibiotic exposure within 90 days. The mean CCI of the enrolled patients was 3.4 ± 1.6. Notably, all patients underwent invasive catheter procedures, among which 77.8% (28/36), 91.7% (33/36), 38.9% (14/36), 86.1% (31/36) and 30.6% (11/36) of patients had central intravenous catheters, urinary catheters, endotracheal tubes, gastrostomy tubes, and drainage tubes, respectively. Furthermore, ten patients experienced metastatic infections. Thirty-two patients (88.9%, 32/36) were identified as HAI, and three (8.3%, 3/36) patients were associated with HCAI. The median SOFA score of the enrolled patients was 4 (percentiles 25 = 1, percentiles 75 = 9). Fifteen patients (41.7%, 15/36) presented with poor prognosis. Specifically, 13 patients died within 30 days, and two patients withheld life-sustaining therapy (Table 1).

**Table 1:** Clinical Characteristics of the Patients with ST11 *Klebsiella pneumoniae* Infection

Patient^a^	Sex	Age	Department	Specimen	Collection date^b^	Underlying diseases							Antibiotics exposure within 90 days	CCI
						**Diabetes**	**Pulmonary disease**	**Cardiovascular disease**	**Cerebrovascular disease**	**Digestive disease**	**Urinary disease**	**Cancer**		
P1	m	62	emergency	sputum	2020/7/23	N	N	Y	Y	Y	N	N	Y	3
P2	m	90	Geriatric Ward	sputum	2020/11/10	N	N	Y	Y	Y	Y	N	Y	5
P3	m	68	ICU	sputum	2020/11/5	N	N	Y	Y	Y	Y	N	Y	5
P4	f	90	emergency	Urine	2020/4/23	N	N	Y	Y	N	N	Y	Y	3
P5	m	81	emergency	sputum	2018/9/25	Y	Y	Y	N	N	Y	N	Y	4
P6	f	93	emergency	sputum	2020/7/19	Y	Y	Y	Y	N	N	N	Y	4
P7	f	71	orthopedics department	Fluid	2020/12/3	N	N	Y	Y	N	N	N	Y	2
P8	f	83	ICU	sputum	2020/10/15	Y	Y	Y	Y	N	N	N	Y	4
P9	m	66	emergency	sputum	2018/9/11	N	N	N	N	Y	N	N	Y	2
P10	f	80	emergency	Urine	2020/11/5	N	N	Y	Y	N	Y	N	Y	3
P11	m	84	emergency	Broncho-alveolar lavage	2020/10/30	Y	N	Y	N	N	Y	N	Y	3
P12	m	75	emergency	Blood	2020/9/8	Y	N	Y	N	Y	Y	N	Y	5
P13	m	84	emergency	sputum	2020/12/7	Y	Y	Y	Y	Y	Y	N	Y	6
P14	f	67	emergency	sputum	2020/10/26	Y	N	Y	N	Y	Y	N	Y	4
P15	f	83	ICU	sputum	2020/9/28	N	N	Y	N	N	N	Y	Y	7
P16	m	85	ICU	sputum	2020/11/4	N	N	Y	Y	N	Y	N	Y	3
P17	m	61	general surgery department	Drainage	2020/11/24	N	N	Y	N	N	Y	N	Y	2
P18	m	85	ICU	Urine	2020/10/20	Y	N	Y	Y	Y	N	N	Y	4
P19	m	65	emergency	sputum	2020/10/21	Y	N	Y	Y	N	N	N	Y	3
P20	f	83	emergency	Urine	2020/5/3	N	N	Y	Y	N	Y	N	Y	3
P21	m	92	ICU	sputum	2018/9/13	N	Y	Y	N	N	N	N	Y	2
P22	f	29	ICU	Throat	2020/9/5	N	N	N	N	N	N	N	Y	0
P23	f	57	emergency	Urine	2020/3/22	Y	N	Y	Y	N	Y	Y	Y	6
P24	f	84	emergency	sputum	2020/12/5	N	N	Y	N	N	N	N	Y	1
P25	f	67	emergency	sputum	2020/9/21	Y	Y	Y	Y	N	N	N	Y	4
P26	f	87	emergency	sputum	2020/9/26	N	N	Y	N	Y	N	Y	Y	5
P27	m	66	ICU	Blood	2020/12/10	N	N	Y	Y	N	N	Y	Y	4
P28	m	83	cardiac surgery ward	Blood	2017/12/11	Y	N	Y	N	N	Y	N	Y	4
P29	m	13	ICU	Urine	2020/12/11	N	N	N	N	N	N	N	Y	0
P30	f	87	emergency	sputum	2020/11/14	Y	N	Y	Y	N	N	N	Y	3
P31	f	83	the branch of the hospital	sputum	2020/10/23	N	N	Y	N	Y	N	N	Y	2
P32	f	86	Geriatric Ward	sputum	2020/10/20	N	N	Y	Y	Y	N	N	Y	3
P33	f	88	outpatient department	Urine	2020/11/18	N	Y	Y	N	N	N	N	Y	2
P34	f	83	Geriatric Ward	Urine	2020/10/16	Y	Y	Y	Y	Y	Y	N	Y	6
P35	m	38	the branch of the hospital	Urine	2020/10/15	N	N	Y	Y	N	N	N	Y	2

Patient^a^	Sex	Age	Department	Specimen	Collection date^b^	Usage of invasive catheters					Metastatic infection	HAI	SOFA	Outcome in 30 days
						**Central intravenous catheter**	**Urinary catheter**	**Endotracheal tube**	**Gastrostomy tube**	**Drainage tube**				
P1	m	62	emergency	sputum	2020/7/23	N	Y	N	Y	Y	N	Y	0	Death^c^
P2	m	90	Geriatric Ward	sputum	2020/11/10	Y	Y	Y	Y	N	N	HCAI	5	Death
P3	m	68	ICU	sputum	2020/11/5	Y	Y	Y	Y	N	N	Y	9	Survive
P4	f	90	emergency	Urine	2020/4/23	Y	Y	Y	Y	N	Y	Y	0	Survive
P5	m	81	emergency	sputum	2018/9/25	N	Y	N	Y	N	N	Y	5	Survive
P6	f	93	emergency	sputum	2020/7/19	Y	Y	N	Y	N	N	Y	0	Survive
P7	f	71	orthopedics department	Fluid	2020/12/3	Y	Y	N	N	Y	Y	Y	0	Survive
P8	f	83	ICU	sputum	2020/10/15	Y	Y	Y	Y	N	N	Y	9	Death
P9	m	66	emergency	sputum	2018/9/11	N	Y	N	Y	N	N	Y	0	Survive
P10	f	80	emergency	Urine	2020/11/5	N	Y	N	Y	N	N	Y	9	Survive
P11	m	84	emergency	Broncho-alveolar lavage	2020/10/30	Y	Y	N	Y	N	N	Y	25	Death
P12	m	75	emergency	Blood	2020/9/8	Y	Y	Y	Y	N	Y	Y	17	Death
P13	m	84	emergency	sputum	2020/12/7	N	N	N	Y	N	N	Y	3	Death
P14	f	67	emergency	sputum	2020/10/26	Y	Y	N	Y	N	N	Y	7	Death
P15	f	83	ICU	sputum	2020/9/28	Y	Y	N	Y	N	N	Y	10	Death
P16	m	85	ICU	sputum	2020/11/4	Y	Y	N	Y	Y	N	Y	2	Survive
P17	m	61	general surgery department	Drainage	2020/11/24	Y	Y	Y	Y	Y	Y	Y	8	Death
P18	m	85	ICU	Urine	2020/10/20	Y	Y	Y	Y	Y	N	Y	3	Survive
P19	m	65	emergency	sputum	2020/10/21	Y	Y	N	Y	Y	N	Y	4	Survive
P20	f	83	emergency	Urine	2020/5/3	Y	Y	N	Y	N	Y	Y	1	Survive
P21	m	92	ICU	sputum	2018/9/13	Y	Y	N	Y	N	N	Y	1	Survive
P22	f	29	ICU	Throat	2020/9/5	Y	Y	Y	Y	N	Y	Y	11	Survive
P23	f	57	emergency	Urine	2020/3/22	Y	Y	Y	Y	Y	Y	Y	3	Survive
P24	f	84	emergency	sputum	2020/12/5	Y	Y	Y	Y	Y	N	Y	7	Death
P25	f	67	emergency	sputum	2020/9/21	Y	Y	N	N	Y	Y	Y	1	Death^c^
P26	f	87	emergency	sputum	2020/9/26	Y	Y	N	Y	N	N	Y	14	Death
P27	m	66	ICU	Blood	2020/12/10	Y	Y	Y	N	N	N	Y	26	Death
P28	m	83	cardiac surgery ward	Blood	2017/12/11	Y	Y	Y	Y	N	N	Y	15	Death
P29	m	13	ICU	Urine	2020/12/11	Y	Y	Y	Y	Y	N	Y	8	Survive
P30	f	87	emergency	sputum	2020/11/14	N	Y	N	Y	N	N	HCAI	6	Death
P31	f	83	the branch of the hospital	sputum	2020/10/23	N	N	N	Y	N	N	Y	0	Survive
P32	f	86	Geriatric Ward	sputum	2020/10/20	Y	Y	N	N	N	Y	Y	3	Survive
P33	f	88	outpatient department	Urine	2020/11/18	Y	Y	N	Y	Y	N	HCAI	4	Survive
P34	f	83	Geriatric Ward	Urine	2020/10/16	Y	Y	N	Y	N	Y	Y	2	Survive
P35	m	38	the branch of the hospital	Urine	2020/10/15	Y	Y	Y	Y	N	N	Y	4	Survive

*Abbreviations*: *Y* Yes, *N* No, *CCI* Charlson comorbidity index, *HAI* Hospital-acquired infection, *HCAI* Healthcare-associated infection, *SOFA* Sequential organ failure assessment

^a^ The patient information of one isolate was missed, ^b^ If several strains were isolated from the same patient, the first strain collection date was recorded, ^c^ Withheld life-sustained treatment

### Subtyping, antimicrobial resistance and virulence profiles

All Kp strains were identified as ST11 (*gapA3-infB3-mdh1-pgi1-phoE1-rpoB1-tonB4*). Twenty-eight strains were identified as hvKp. Most of the Kp strains (98.3%, 57/58) carried *yersiniabactin. IucA* was positive in 28 strains (48.3%, 28/58), and 17 isolates (29.3%, 17/58) harbored *rmpA*. Sixteen isolates contained *peg-344* (27.9%, 16/58). Only one strain was associated with *iroB*. Surprisingly, none of the strains harbored *colibactin* or *rmpA2.* The common key virulence genotype was *iucA* + *peg-344* + *rmpA.* For the serotype, KL64 was positive in 27 strains (46.6%, 27/58), and KL47 was positive in 28 isolates (48.3%, 28/58). The other three strains presented with KL25.

All the ST11 Kp strains exhibited resistance against more than 3 classes of antimicrobial agents and presented with MDR, especially CR, phenotypes. More specifically, all the ST11 strains were resistant to TZP, CAZ, IPM, MEM, ETP and FEP. Additionally, only one isolate showed an intermediary for the LVX and CSL. Seven strains (12.1%, 7/58) were susceptible to AMK. Fortunately, 58.6% (34/58) of the strains were susceptible to SXT, and the 30 isolates were associated with a nonresistance phenotype for MNO (Additional file 1: Table S1).

**Fig. 1:**
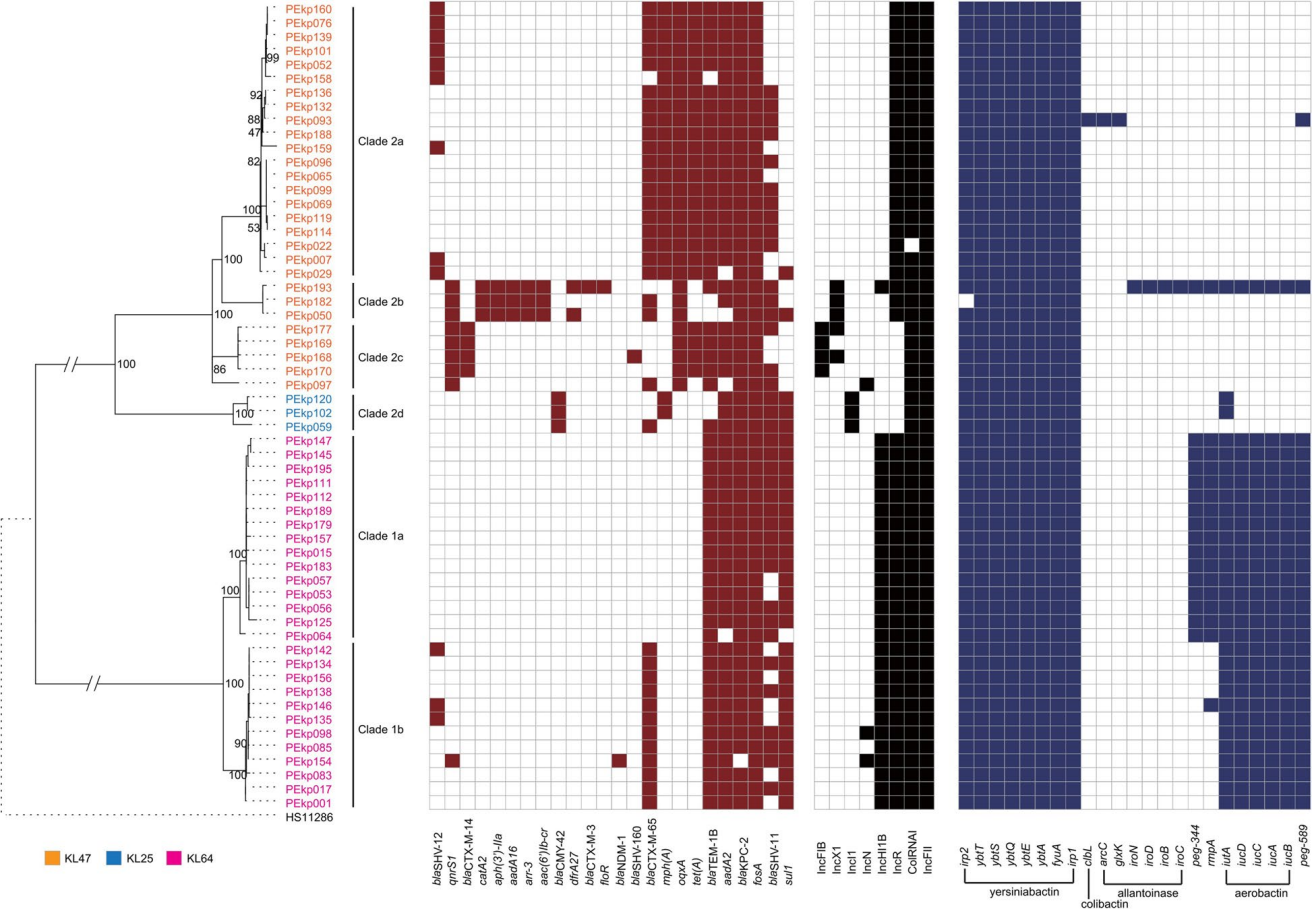
Phylogenetic tree of the ST11 *Klebsiella pneumoniae* strains. Orange: KL47 Blue: KL25. Pink: KL64

All the ST11 strains harbored *fosA* before fosfomycin exposure. Only one Kp strain did not present with *bla*_*KPC-2.*_ Among the *bla*_*CTX-M-like*_ positive strains, most of the strains (60.3%, 35/58) were associated with *bla*_*CTX-M-65*_*,* distributed in CR-hvKp (12 strains) and CR-cKp (23 strains)*.* The strains that harbored *bla*_*CTX-M-14*_ (4 strains) and *bla*_*CTX-M-3*_ (1 strain) belonged to cKp*.* Even worse, *bla*_*NDM-1*_ emerged in the hospital. Another common ESBL, *bla*_*TEM-1B*_, existed in 53 isolates. Furthermore, twenty-four strains carried *tet(A)* (Fig. 1).

Combined with virulence and AST, 28 strains were defined as CR-hvKp, and 30 isolates were CR-cKp. The common genomic characteristics of CR-hvKp presented with KL64 + *iucA* + *peg-344* + *bla*_*KPC-2*_ + *bla*_*TEM-1B,*_ whereas CR-cKp frequently harbored KL47 + *bla*_*KPC-2*_ + *bla*_*TEM-1B*_ + *tet(A)* + *bla*_*CTX-M-65.*_

All strains harbored the IncFII plasmid replicon, and 98.3% (57/58) contained the ColRNAI plasmid replicon. Fifty strains (86.2%, 50/58) carried IncR plasmid replicons, and 48.3% (28/58) were positive for IncHI1B plasmid replicons. More than four types of replicons were present in 3/58 (5.2%) isolates (Fig. 1).

### Phylogenetic relationships and distribution of the CRKP

In total, 1,325 high quality SNPs were identified and applied for phylogenetic analysis (Fig. 1). Among all the ST11 CRKP strains, two distinguished clades were identified. Clade 1 contained 27 strains belonging to the CR-hvKp and KL64 serotypes, most of which were collected from the emergency department (14 isolates) and ICU (6 isolates). Clade 1 consisted of two clusters, clade 1a and clade 1b. Notably, all 15 strains of clade 1a harbored *peg-344* + *rmpA*, while only one strain of clade 1b (PEkp146) possessed *rmpA* and no strain possessed *peg-344*. Additionally, all 12 isolates of clade 1b harbored *bla*_*CTX-M-65*_, which wasn’t present in clade 1a. Clade 2 was comprised of 31 isolates, which contained four clusters, clade 2a, clade 2b, clade 2c and clade 2d, and could be separated into two large branches. Clades 2a, 2b and 2c belonged to the KL47 branch, and clade 2d belonged to the KL25 branch. Clade 2d comprised of three isolates, which possessed *bla*_*CMY-42*_ and carried IncI1 plasmid replicon. Three strains of Clade 2b harbored *catA2, aph(3’)-IIa, aadA1, arr-3* and *aac(6’)Ib-cr*. All isolates of clade 1 were hvKp. Notably, all strains of clade 2 were identified as CR-cKp, except only one strain, PEkp193, which might become CR-hvKp from CR-cKp during within-host evolution.

### Transmission route of CR-hvKp and CR-cKp

**Fig. 2:**
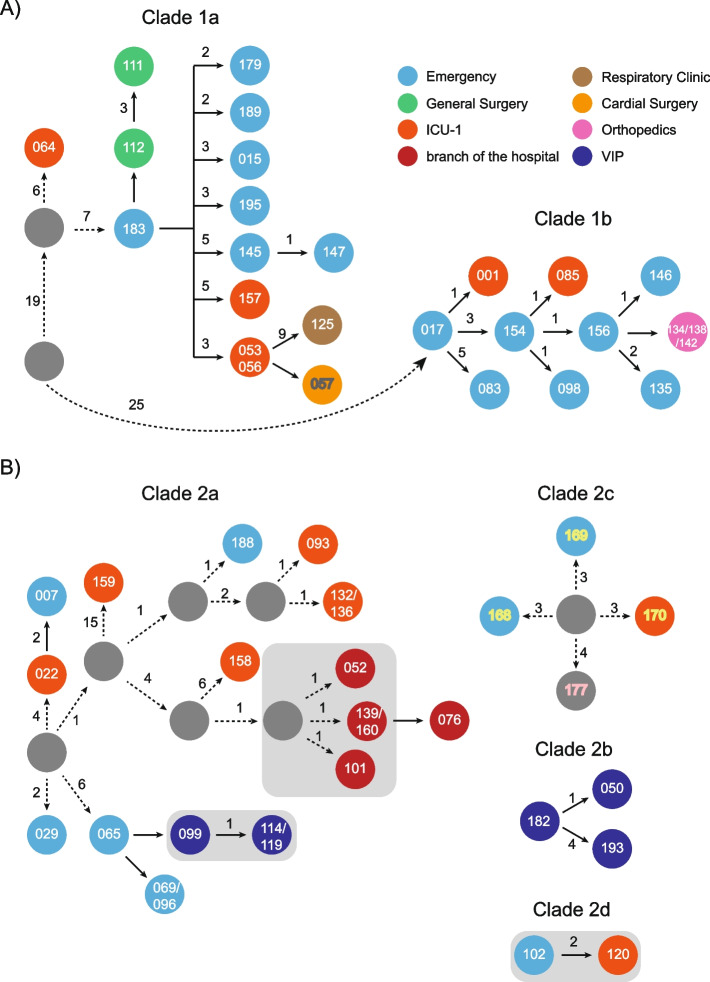
Evolutionary and transmission route of the isolated *Klebsiella pneumoniae* strains. **A** clade 1 strains. **B** clade 2 strains. Gray circle, strains that were not captured during transmission in this study. The numbers on the arrows are SNPs. Gray shadow, strains were from the same patient. Different Colors of strain number represent different collection date. White: 2020; Yellow: 2018; Gray, 2017; Pink: Missing

After combining the SNPs with epidemiological information, we reconstructed the potential transmission routes among these patients (Fig. 2). It is estimated that the hvKp ancestor might have separated from an ancestor and then spread into the emergency department and ICU (Fig. 2A). Briefly, the common ancestor of Clade 1 evolved into two main subclones (PEkp183 and PEkp017), which resulted in circulation within the emergency department and then spread to the outpatient department and other departments. Importantly, the PEkp125 strain was isolated from respiratory clinics and triggered HCAI.

Most of the strains (34.5%, 20/58) in Clade 2 were clustered within Clade 2a (Fig. 2B). The common ancestor of clade 2a underwent slight changes in SNPs during inter- and intrahost evolution, suggesting that the MDR ST11 Kp strains are circulating within the ICU and emergency department. More importantly, MDR-cKp has been transmitted into the branch of the hospital, alarming that enhancing surveillance is essential. Additionally, PEkp099 resulted in HCAI, suggesting that ST11 CR-cKp might successfully transmit into the healthcare-associated institution. All strains from the same patients belonged to the same clade, which presented high similarity in SNPs. Notably, although tiny differences in SNPs were shown among PEkp050, PEkp182 and PEkp193 within the same host, PEkp193 acquired *iucA* + *peg-344* + *peg-589* + *rmpA* and subsequently conferred CR-hvKp.

## Discussion

In this study, we characterized the ST11 genome during inter- and intrahost evolution and transmission in the hospital. CR-hvKp presented with KL64, and CR-cKp harboring KL47 was distinctly distributed into two major clades, resulting in prolonged circulation within the ICU and emergency department, and might subsequently be transmitted into healthcare-associated institutes, indicating that genomic surveillance should be enhanced.

A recent epidemiological survey in China confirmed the rapid rise of MDR strains attributed to the ST11 clone [1, 25], which is a common cause of hospital infection outbreaks [26, 27]. Different from the classical hvKp (i.e., ST23), the loss of the “thick wall” of cKp enhanced the acquisition of external genetic elements [28]. Notably, all the ST11 strains were MDR and CR in our study, regardless of cKp or hvKp. A previous study in our country reported an outbreak due to the hypermucoviscous ST11 CRKP with an unclear serotype that occurred in China by PFGE [29] and then was the fatal outbreak of ST11-CR-hvKp, which was confirmed by whole genome sequencing and an in vivo model [17]. In this study, we reported that ST11 CRKP has evolved into KL64-CR-hvKp and KL47-CR-cKp, with parallel spreading in the hospital.

A previous study reported that the pandemic spread of *bla*_*KPC-2*_ among ST11 in China is mediated by the horizontal genetic element IncFII-like plasmids [30]. Similarly, all the ST11 strains except one in our group harbored IncFII plasmid replications carrying *bla*_*KPC-2*_ and were resistant to carbapenems. One strain, PEkp154, did not harbor *bla*_*KPC-2*_ but presented with a CR phenotype, which might be due to the presence of *bla*_*NDM-1*_. Based on the *bla*_*KPC-2*_ genetic context, ST11 frequently presented with MDR or CR phenotypes. Importantly, ST11, the epidemic sequence type in China, tends to take on various virulence-associated genes, becoming MDR-hvKp [11, 16]. A previous study reported that the CRKP ST11 clone with coproduction of *bla*_*CTX-M-65*_ and *bla*_*KPC-2*_ disseminated in the ICU and suggested that the CRKP presented with hypermucoviscosity [31]. Even more disturbing is that a fatal outbreak due to CR-ST11-hvKp occurred in the ICU, resulting in poor prognosis in all patients [17]. In the study, almost all the ST11 CR-hvKp strains coharbored *bla*_*KPC-2*_ and *bla*_*TEM-1B*_, and approximately half of them also carried *bla*_*CTX-M-65*_*,* suggesting that the trend of convergence of the AMR and virulence genes is forming. Moreover, our previous study demonstrated that various virulence-associated genes and AMR genes converged within the ST11 clone during interhost evolution and transmission [14]. More importantly, CAI caused by MDR-ST11-hvKp was also detected [14]. Whole-genome sequencing may provide improved resolution to define transmission pathways and characterize outbreaks, instructing the prevention and treatment of infection control. Previous study reported that WGS could effectively investigate MRSA or CR-hvKp outbreak and timely enhance intervention of infection control, liking disinfection and unoccupied period [17, 32–34]. In the study, CR-hvKp might be successfully transmitted within the hospital and/or healthcare institutes, and CR-cKp has diffused into two branches, suggesting that genomic surveillance is essential and should cover healthcare institutes.

Our previous study demonstrated that cKp, primary ST11, acquiring the pVir-like plasmid (*iucA* + *rmpA2*) or virulence-associated genes is the common reason for shaping MDR-hvKp, resulting in a major shift in hvKp epidemiology [11]. A study conducted in multiple centers in Japan also demonstrated that hvKp are frequently involved in HCAI and HAI [35]. ST11-KL64 still frequently presents with *bla*_*KPC-2*_ [36]. Additionally, ST11-KL64 CR-hvKp could rapidly gain and lose genes during interhospital transmission [37]. Recently, an important reason for enhanced transmission is that the fused plasmid converged with AMR and virulence genes [38]. However, the key virulence gene, *iucA*, was predominant within the ST11 CR-hvKp group, but none of the strains possessed *rmpA2*, suggesting that new plasmids might emerge and may have a tendency to spread. Long-read sequencing should be used for further study.

The KL64 and KL47 serotypes were common in the ST11 clone [11]. Interestingly, a previous study conducted in a single center in Zhe Jiang concluded that the ST11-KL64 clone is replacing ST11-K47 due to a new fused plasmid and that the ST11-KL64 capsule type may be more virulent [15]. Our previous study reported that KL47 was the dominant serotype in ST11 [14]. In this study, KL64 and KL47 accounted for nearly half. Moreover, all the KL64-positive strains carried various key virulence-associated genes, whereas only one KL47 strain possessed *iroB* + *peg-344* + *iucA* + *rmpA*, which might be similar to the pLVPK-like plasmid [3]. Interestingly, 8 patients (50%, 8/16) with KL64-ST11 Kp infections presented with poor outcomes, whereas 6 patients (35.3%, 6/17) who had ST11-KL47 Kp died.

The main limitation of our study was that it was a retrospective study conducted at a single center. Furthermore, the number of strains was small, and they were randomly selected. A larger sample size originating from multiple centers is needed to deeply understand the genetic epidemiology. Additionally, long-read sequencing may assist us in fully characterizing the virulence-associated plasmid context. Last, an animal model should be selected to identify the virulence phenotype.

In conclusion, KL64-CR-hvKp and KL47-CR-cKp within ST11 were spread in parallel in the hospital. Importantly, ST11-CR-hvKp presenting with KL64 might successfully transmit within the hospital and/or healthcare institutes, and ST11-CR-cKp associated with KL47 spread to the two branches of the hospital, suggesting that ongoing surveillance of the endemic ST11 clone is critical.

## Supplementary Information

**Additional file 1:** **Figure S1.** Collection date of these ST11-Kp strains. **Figure S2.** Specimen type of these ST11-Kp strains. **Table S1.** Antimicrobial resistance profile of these ST11-Kp strains.

## Data Availability

All ST11 *K. pneumoniae* genome sequences have been deposited in the NCBI Sequencing Read Archive database under the accession number PRJNA806574. The datasets used and/or analyzed during the current study are available from the corresponding author on reasonable request.
