# Supplementary material for: Convergence of two serotypes within the epidemic ST11 KPC-producing Klebsiella pneumoniae creates the “Perfect Storm” in a teaching hospital
Source: BMC Genomics. 2022 Oct 7;23:693. doi: 10.1186/s12864-022-08924-8 (PMC9540996; doi:10.1186/s12864-022-08924-8)
Supplement: Supplementary file 1 — Additional file 1: Figure S1. Collection date of these ST11-Kp strains. Figure S2. Specimen type of these ST11-Kp strains. Table S1. Antimicrobial resistance profile of these ST11-Kp strains. [file 12864_2022_8924_MOESM1_ESM.docx]

Supplementary figure 1. Collection date of these ST11-Kp strains.

Supplementary figure 2. Specimen type of these ST11-Kp strains.

BALF: bronchoalveolar lavage fluid.

Supplementary table 1. Antimicrobial resistance profile of these ST11-Kp strains.

| **No.** | **TZP** | | **CAZ** | | **IPM** | | **MEM** | | **LVX** | | **FEP** | | **SXT** | | **AMK** | | **MNO** | | **ETP** | | **CSL** | |
| --- | --- | --- | --- | --- | --- | --- | --- | --- | --- | --- | --- | --- | --- | --- | --- | --- | --- | --- | --- | --- | --- | --- |
| PEkp001 | >=128 | R | >=64 | R | >=16 | R | 6 | R | >=8 | R | >=32 | R | <=20 | S | >=64 | R | 16 | S | >=8 | R | >=64 | R |
| PEkp007 | >=128 | R | >=64 | R | >=16 | R | 6 | R | >=8 | R | >=32 | R | >=320 | R | >=64 | R | 13 | I | >=8 | R | >=64 | R |
| PEkp015 | >=128 | R | >=64 | R | >=16 | R | >=16 | R | >=8 | R | >=32 | R | <=20 | S | >=64 | R | 4 | S | >=8 | R | >=64 | R |
| PEkp017 | >=128 | R | >=64 | R | >=16 | R | 6 | R | >=8 | R | >=32 | R | <=20 | S | >=64 | R | 20 | S | >=8 | R | >=64 | R |
| PEkp022 | >=128 | R | >=64 | R | >=16 | R | 6 | R | >=8 | R | >=32 | R | >=320 | R | >=64 | R | 14 | I | >=8 | R | >=64 | R |
| PEkp029 | >=128 | R | >=64 | R | >=16 | R | 6 | R | >=8 | R | >=32 | R | >=320 | R | >=64 | R | 12 | R | >=8 | R | >=64 | R |
| PEkp050 | >=128 | R | >=64 | R | >=16 | R | 6 | R | >=8 | R | >=32 | R | >=320 | R | >=64 | R | 10 | R | >=8 | R | >=64 | R |
| PEkp052 | >=128 | R | >=64 | R | >=16 | R | 6 | R | >=8 | R | >=32 | R | <=20 | S | <=2 | S | >=16 | R | 6 | R | >=64 | R |
| PEkp053 | >=128 | R | >=64 | R | >=16 | R | >=16 | R | >=8 | R | >=32 | R | <=20 | S | >=64 | R | >=16 | R | 6 | R | >=64 | R |
| PEkp056 | >=128 | R | >=64 | R | >=16 | R | >=16 | R | 4 | R | >=32 | R | 160 | R | >=64 | R | 4 | S | 6 | R | 16 | I |
| PEkp057 | >=128 | R | >=64 | R | >=16 | R | >=16 | R | >=8 | R | >=32 | R | <=20 | S | >=64 | R | >=16 | R | 6 | R | >=64 | R |
| PEkp059 | >=128 | R | >=64 | R | >=16 | R | 6 | R | >=8 | R | >=32 | R | 160 | R | >=64 | R | 20 | S | >=8 | R | >=64 | R |
| PEkp064 | >=128 | R | >=64 | R | >=16 | R | 6 | R | >=8 | R | >=32 | R | <=20 | S | >=64 | R | 21 | S | >=8 | R | >=64 | R |
| PEkp065 | >=128 | R | >=64 | R | >=16 | R | 6 | R | >=8 | R | >=32 | R | >=320 | R | >=64 | R | 15 | I | >=8 | R | >=64 | R |
| PEkp069 | >=128 | R | >=64 | R | >=16 | R | >=16 | R | >=8 | R | >=32 | R | >=320 | R | >=64 | R | >=16 | R | 6 | R | >=64 | R |
| PEkp076 | >=128 | R | >=64 | R | >=16 | R | 11 | R | >=8 | R | >=32 | R | 40 | S | >=64 | R | 12 | R | >=8 | R | >=64 | R |
| PEkp083 | >=128 | R | >=64 | R | >=16 | R | >=16 | R | >=8 | R | >=32 | R | <=20 | S | >=64 | R | >=16 | R | 6 | R | >=64 | R |
| PEkp085 | >=128 | R | >=64 | R | >=16 | R | >=16 | R | >=8 | R | >=32 | R | <=20 | S | >=64 | R | >=16 | R | 6 | R | >=64 | R |
| PEkp093 | >=128 | R | >=64 | R | >=16 | R | >=16 | R | >=8 | R | >=32 | R | >=320 | R | >=64 | R | >=16 | R | >=8 | R | >=64 | R |
| PEkp096 | >=128 | R | >=64 | R | >=16 | R | >=16 | R | >=8 | R | >=32 | R | >=320 | R | >=64 | R | >=16 | R | 6 | R | >=64 | R |
| PEkp097 | >=128 | R | >=64 | R | >=16 | R | >=16 | R | >=8 | R | >=32 | R | <=20 | S | >=64 | R | 2 | S | 6 | R | >=64 | R |
| PEkp098 | >=128 | R | >=64 | R | >=16 | R | 6 | R | >=8 | R | >=32 | R | 40 | S | >=64 | R | 16 | S | >=8 | R | >=64 | R |
| PEkp099 | >=128 | R | >=64 | R | >=16 | R | >=16 | R | >=8 | R | >=32 | R | >=320 | R | >=64 | R | >=16 | R | 6 | R | >=64 | R |
| PEkp101 | >=128 | R | >=64 | R | >=16 | R | >=16 | R | >=8 | R | >=32 | R | 160 | R | 8 | S | >=16 | R | >=8 | R | >=64 | R |
| PEkp102 | >=128 | R | >=64 | R | >=16 | R | 6 | R | >=8 | R | >=32 | R | >=320 | R | <=2 | S | 18 | S | >=8 | R | >=64 | R |
| PEkp111 | >=128 | R | >=64 | R | >=16 | R | >=16 | R | >=8 | R | >=32 | R | <=20 | S | >=64 | R | >=16 | R | 6 | R | >=64 | R |
| PEkp112 | >=128 | R | >=64 | R | >=16 | R | >=16 | R | >=8 | R | >=32 | R | <=20 | S | >=64 | R | >=16 | R | 6 | R | >=64 | R |
| PEkp114 | >=128 | R | >=64 | R | >=16 | R | >=16 | R | >=8 | R | >=32 | R | >=320 | R | >=64 | R | >=16 | R | 6 | R | >=64 | R |
| PEkp119 | >=128 | R | >=64 | R | >=16 | R | >=16 | R | >=8 | R | >=32 | R | >=320 | R | >=64 | R | >=16 | R | 6 | R | >=64 | R |
| PEkp120 | >=128 | R | >=64 | R | >=16 | R | >=16 | R | >=8 | R | >=32 | R | >=320 | R | <=2 | S | 8 | I | 6 | R | >=64 | R |
| PEkp125 | >=128 | R | >=64 | R | >=16 | R | 6 | R | >=8 | R | >=32 | R | <=20 | S | >=64 | R | 18 | S | >=8 | R | >=64 | R |
| PEkp132 | >=128 | R | >=64 | R | >=16 | R | >=16 | R | >=8 | R | >=32 | R | >=320 | R | >=64 | R | >=16 | R | >=8 | R | >=64 | R |
| PEkp134 | >=128 | R | >=64 | R | >=16 | R | >=16 | R | >=8 | R | >=32 | R | <=20 | S | >=64 | R | 8 | I | 6 | R | >=64 | R |
| PEkp135 | >=128 | R | >=64 | R | >=16 | R | >=16 | R | >=8 | R | >=32 | R | <=20 | S | >=64 | R | 8 | I | 6 | R | >=64 | R |
| PEkp136 | >=128 | R | >=64 | R | >=16 | R | >=16 | R | >=8 | R | >=32 | R | >=320 | R | >=64 | R | >=16 | R | >=8 | R | >=64 | R |
| PEkp138 | >=128 | R | >=64 | R | >=16 | R | >=16 | R | >=8 | R | >=32 | R | <=20 | S | >=64 | R | 8 | I | 6 | R | >=64 | R |
| PEkp139 | >=128 | R | >=64 | R | >=16 | R | >=16 | R | >=8 | R | >=32 | R | 40 | S | >=64 | R | >=16 | R | 10 | R | >=64 | R |
| PEkp142 | >=128 | R | >=64 | R | >=16 | R | >=16 | R | >=8 | R | >=32 | R | <=20 | S | >=64 | R | >=16 | R | 6 | R | >=64 | R |
| PEkp145 | >=128 | R | >=64 | R | >=16 | R | >=16 | R | >=8 | R | >=32 | R | <=20 | S | >=64 | R | 8 | I | 6 | R | >=64 | R |
| PEkp146 | >=128 | R | >=64 | R | >=16 | R | >=16 | R | >=8 | R | >=32 | R | <=20 | S | >=64 | R | 8 | I | 6 | R | >=64 | R |
| PEkp147 | >=128 | R | >=64 | R | >=16 | R | >=16 | R | >=8 | R | >=32 | R | <=20 | S | >=64 | R | 8 | I | 6 | R | >=64 | R |
| PEkp154 | >=128 | R | >=64 | R | >=16 | R | >=16 | R | >=8 | R | >=32 | R | 40 | S | >=64 | R | >=16 | R | 6 | R | >=64 | R |
| PEkp156 | >=128 | R | >=64 | R | >=16 | R | >=16 | R | 4 | R | >=32 | R | <=20 | S | 4 | S | 4 | S | 6 | R | >=64 | R |
| PEkp157 | >=128 | R | >=64 | R | >=16 | R | >=16 | R | >=8 | R | >=32 | R | <=20 | S | >=64 | R | 4 | S | 6 | R | >=64 | R |
| PEkp158 | >=128 | R | >=64 | R | >=16 | R | >=16 | R | >=8 | R | >=32 | R | 40 | S | <=2 | S | >=16 | R | 6 | R | >=64 | R |
| PEkp159 | >=128 | R | >=64 | R | >=16 | R | >=16 | R | >=8 | R | >=32 | R | <=20 | S | >=64 | R | >=16 | R | 6 | R | >=64 | R |
| PEkp160 | >=128 | R | >=64 | R | >=16 | R | >=16 | R | >=8 | R | >=32 | R | <=20 | S | >=64 | R | >=16 | R | 6 | R | >=64 | R |
| PEkp168 | >=128 | R | >=64 | R | >=16 | R | 13 | R | 1 | I | >=32 | R | <=20 | S | >=64 | R | 12 | R | >=8 | R | >=64 | R |
| PEkp169 | >=128 | R | >=64 | R | >=16 | R | 6 | R | >=8 | R | >=32 | R | >=320 | R | >=64 | R | 16 | S | >=8 | R | >=64 | R |
| PEkp170 | >=128 | R | >=64 | R | >=16 | R | 6 | R | >=8 | R | >=32 | R | >=320 | R | >=64 | R | 14 | I | >=8 | R | >=64 | R |
| PEkp179 | >=128 | R | >=64 | R | >=16 | R | 6 | R | >=8 | R | >=32 | R | <=20 | S | >=64 | R | 14 | I | >=8 | R | >=64 | R |
| PEkp182 | >=128 | R | >=64 | R | >=16 | R | 6 | R | >=8 | R | >=32 | R | >=320 | R | >=64 | R | 19 | S | >=8 | R | >=64 | R |
| PEkp183 | >=128 | R | >=64 | R | >=16 | R | 6 | R | >=8 | R | >=32 | R | <=20 | S | >=64 | R | 15 | I | >=8 | R | >=64 | R |
| PEkp188 | >=128 | R | >=64 | R | >=16 | R | 6 | R | >=8 | R | >=32 | R | >=320 | R | >=64 | R | 6 | R | >=8 | R | >=64 | R |
| PEkp189 | >=128 | R | >=64 | R | >=16 | R | 6 | R | >=8 | R | >=32 | R | <=20 | S | >=64 | R | 6 | R | >=8 | R | >=64 | R |
| PEkp193 | >=128 | R | >=64 | R | >=16 | R | 6 | R | >=8 | R | >=32 | R | >=320 | R | <=2 | S | 14 | I | >=8 | R | >=64 | R |
| PEkp195 | >=128 | R | >=64 | R | >=16 | R | 6 | R | >=8 | R | >=32 | R | <=20 | S | >=64 | R | 16 | S | >=8 | R | >=64 | R |
| PEkp177 | >=128 | R | >=64 | R | >=16 | R | 6 | R | >=8 | R | >=32 | R | >=320 | R | >=64 | R | 14 | I | >=8 | R | >=64 | R |

Imipenem (IPM); Meropenem (MEM); Ertapenem (ETP); Minocycline (MNO); Amikacin (AMK); Cefepime (FEP); Ceftazidime (CAZ); Levofloxacin (LVX); Piperacillin/tazobactam (TZP); Cefoperazone/sulbactam (CSL); trimethoprim/sulfamethoxazole (SXT).
